# The Impact of Heating, Ventilation, and Air-Conditioning Design Features on the Transmission of Viruses, Including SARS-CoV-2: Overview of Reviews

**DOI:** 10.2196/37232

**Published:** 2022-12-23

**Authors:** Gail M Thornton, Emily Kroeker, Brian A Fleck, Lexuan Zhong, Lisa Hartling

**Affiliations:** 1 Department of Mechanical Engineering Faculty of Engineering University of Alberta Edmonton, AB Canada; 2 Department of Pediatrics Faculty of Medicine & Dentistry University of Alberta Edmonton, AB Canada

**Keywords:** COVID-19, public health, epidemiology, outbreak, pandemic, environment, literature review, virus transmission, ventilation, coronavirus

## Abstract

**Background:**

The COVID-19 or SARS-CoV-2 outbreak was declared a pandemic by the World Health Organization in March 2020. Almost 2 years later (early February 2022), the World Health Organization reported over 383 million cases of the disease caused by the virus, with over 5.6 million deaths worldwide. Debate regarding the routes of transmission was substantial early in the pandemic; however, airborne transmission emerged as an important consideration. Infectious airborne agents can spread within the built environment through heating, ventilation, and air-conditioning (HVAC) systems. Multiple features of HVAC systems can influence transmission (eg, ventilation, filtration, UV radiation, and humidity). Understanding how HVAC features influence airborne transmission is critical to mitigate the spread of infectious agents.

**Objective:**

Given the airborne transmission of SARS-CoV-2, an overview of reviews was conducted to understand what is already known from the scientific literature about how virus transmission may be affected by HVAC design features in the built environment.

**Methods:**

Ovid MEDLINE and Compendex were searched from inception to January 2021. Two reviewers independently screened the titles, abstracts, and full text of potentially relevant reviews, using a priori inclusion criteria: systematic reviews examining the effects of HVAC design features on virus transmission. Two reviewers independently assessed the methodological quality using AMSTAR2.

**Results:**

Searching identified 361 citations, of which 45 (12.5%) were potentially relevant and 7 (2%) were included. Reviews were published between 2007 and 2021 and included 47 virus studies. Two earlier reviews (2007 and 2016) of 21 studies found sufficient evidence that mechanical ventilation (airflow patterns and ventilation rates) plays a role in airborne transmission; however, both found insufficient evidence to quantify the minimum mechanical ventilation requirements. One review (2017) of 9 studies examining humidity and indoor air quality found that influenza virus survival was lowest between 40% and 80% relative humidity; the authors noted that ventilation rates were a confounding variable. Two reviews (2021) examined mitigation strategies for coronavirus transmission, finding that transmission decreased with increasing temperature and relative humidity. One review (2020) identified 14 studies examining coronavirus transmission in air-conditioning systems, finding that HVAC systems played a role in virus spread during previous coronavirus outbreaks. One review (2020) examined virus transmission interventions in public ground transportation, finding ventilation and filtration to be effective.

**Conclusions:**

Seven reviews synthesizing 47 studies demonstrated a role for HVAC in mitigating airborne virus transmission. Ventilation, humidity, temperature, and filtration can play a role in the viability and transmission of viruses, including coronaviruses. Recommendations for minimum standards were not possible owing to few studies investigating a given HVAC parameter. This overview examining HVAC design features and their effects on the airborne transmission of viruses serves as a starting point for future systematic reviews and identifying priorities for primary research.

## Introduction

### Background

The COVID-19 or SARS-CoV-2 outbreak, first detected in Wuhan, China, was characterized as a pandemic by the World Health Organization (WHO) in March 2020 [1]. Almost 2 years later (early February 2022), the WHO reported over 383 million cases of the disease (COVID-19) caused by the virus (SARS-CoV-2), with over 5.6 million deaths worldwide [2]. Early in the pandemic, there were conflicting views and debate about the routes of transmission [3-6]. Several recent reviews of the scientific literature have identified evidence indicating airborne transmission, which could be particularly problematic in confined and crowded indoor spaces [7-9]. Public health recommendations acknowledge airborne transmission as important and advise to maximize ventilation; ensure proper maintenance and functioning of heating, ventilation, and air-conditioning (HVAC) systems; and increase the use of fresh air where possible [10].

Airborne transmission occurs as a result of bioaerosols (biological particles suspended in air) staying aloft longer because of their small size and, therefore, traveling further because of air currents [3]. Several possible mechanisms of airborne coronavirus transmission exist, including 1) bioaerosol generation by infectious persons through coughing, sneezing, breathing, and talking, which remain airborne for a period of hours to days; 2) short- to long-range transport through HVAC systems and subsequent inhalation of bioaerosols by other people; and 3) airborne transport of bioaerosols to surfaces (or the contamination of surfaces by physical contact), followed by resuspension, inhalation, or contact with surfaces [11,12].

### Prior Work

Previous research demonstrated that infectious airborne bioaerosols spread to other spaces via HVAC systems [12,13]. Multiple features within HVAC systems may influence transmission, including ventilation (eg, ventilation rate, air changes per hour, airflow pattern, and pressurization), filtration (eg, minimum efficiency reporting value rating, filter age, and extent of use), UV radiation (eg, UV power and UV dose), and humidity [12]. Understanding the influences of HVAC systems on airborne transmission in the built environment is critical for building scientists to develop effective engineering control strategies to protect the occupant’s health and well-being and affect timely public health policies. Previous systematic reviews provided a starting point for understanding what is already known from the scientific literature about HVAC systems and the airborne transmission of viruses. A comprehensive synthesis of previous systematic reviews can help identify knowledge gaps, helping to guide and prioritize future primary research. Therefore, we conducted an overview of reviews to identify and synthesize previous systematic reviews on this topic.

## Methods

Standards recommended by the international Cochrane organization for the conduct of an overview of reviews [14] were followed. The research question guiding this work was as follows: what is the current synthesized evidence about the effects of HVAC design features on virus transmission?

### Search Strategy

A research librarian (GMT) conducted searches in Ovid MEDLINE and Compendex from inception to June 2020*,* using concepts related to viruses, transmission, and HVAC. The search was updated in January 2021. The search strategies are presented in Multimedia Appendix 1. The unfiltered search strategies were peer reviewed by 2 librarians (TL and AH), and the filter for systematic reviews in Ovid MEDLINE was provided by a third librarian (LD). The unfiltered search strategies were part of a larger systematic review project that was registered [15], and its protocol is publicly available [16]. The reference lists of the included reviews were screened to identify any other relevant reviews. Conference abstracts and preprints retrieved through the searches were screened to determine whether a full peer-reviewed manuscript was published. The references were managed in EndNote; duplicate records were removed before screening.

### Study Selection

Two reviewers (GMT and LH) independently screened the titles and abstracts of all the citations retrieved from the electronic searches and other sources. Studies were classified as yes, no, or maybe. The first stage of screening was completed in Covidence. We retrieved the full text of all the studies classified as yes or maybe. The same reviewers independently applied the inclusion and exclusion criteria (Multimedia Appendix 2 [16]) to each full-text document and classified the studies as included or excluded. Discrepancies were resolved through discussion between the 2 reviewers. The reasons for excluding studies at the full-text stage were documented.

### Inclusion and Exclusion Criteria

The inclusion and exclusion criteria are detailed in Multimedia Appendix 2. We planned to include systematic reviews published in English that searched for and included primary research studies examining the effects of HVAC design features on the transmission of viruses. The HVAC features of interest were mechanical ventilation (ventilation rate, air change, air exchange, and airflow), filtration (air filtration, filter type, minimum efficiency reporting value rating, filter age and use, pressure drop, holding capacity, replacement, and change frequency), UV germicidal irradiation (power, dose, uniformity of dose, flow rate, bioaerosol inactivation efficiency, and location), and humidity or relative humidity (RH). Inclusion was staged in 2 ways. Our primary interest was viruses, and we excluded those reviews that were not specific to virus. We were initially interested in systematic reviews defined by the international Cochrane organization as reviews that use a predefined, systematic approach and follow standard approaches to search the literature, select studies for inclusion, assess the methodological quality of the included studies, and extract, synthesize, and analyze data from the included studies. As we found few systematic reviews meeting these criteria, we included review articles that satisfied specific requirements for methodological approach and objective. For methodological approach requirements, the authors had to search ≥2 databases, describe inclusion and exclusion criteria, and describe a process for study selection. For objective requirements, the objective of the review had to be related to one of the HVAC design features, namely ventilation, filtration, UV radiation, or humidity.

### Quality Assessment

The methodological quality of the included reviews was assessed using AMSTAR2 [17]. AMSTAR2 is a valid and reliable tool containing 16 items about the methodological conduct of a systematic review [18]. Two authors (GMT and LH) independently assessed the included reviews. Discrepancies were resolved through discussion.

### Data Extraction

The following information was extracted from each review: citation information (eg, authors, year of publication, and country of corresponding author), objectives, search strategy, inclusion and exclusion criteria, settings, population characteristics (as applicable), agent studied (eg, type of virus and bioaerosol), HVAC design features studied, number and characteristics of studies relevant to this overview’s research question, results (as reported by the review authors), and review authors’ conclusions relevant to this overview’s research question. Our primary outcome was the quantitative measure of the association between HVAC design features and virus transmission; however, we extracted any results reported by the review authors that were relevant to our research question. One reviewer (LH) extracted data using a predefined form. A second reviewer (EK) verified the data. Discrepancies were resolved through discussion and by referring to the relevant publication.

### Data Analysis

We anticipated that the included reviews would not have conducted meta-analyses. We planned to present the results in tabular and narrative forms. Tables were created describing the reviews, their results (including any quantitative data of the associations between HVAC features and virus transmission or proxy outcomes) and conclusions, and their methodological quality. A narrative summary of the findings of each review has been provided. We only summarize review findings that were relevant to our research question; for example, if the review included studies of ventilation, humidity, etc, in the outdoor and indoor environments, we only report on studies specific to the indoor (built) environment.

## Results

### Included Reviews

**Figure 1:**
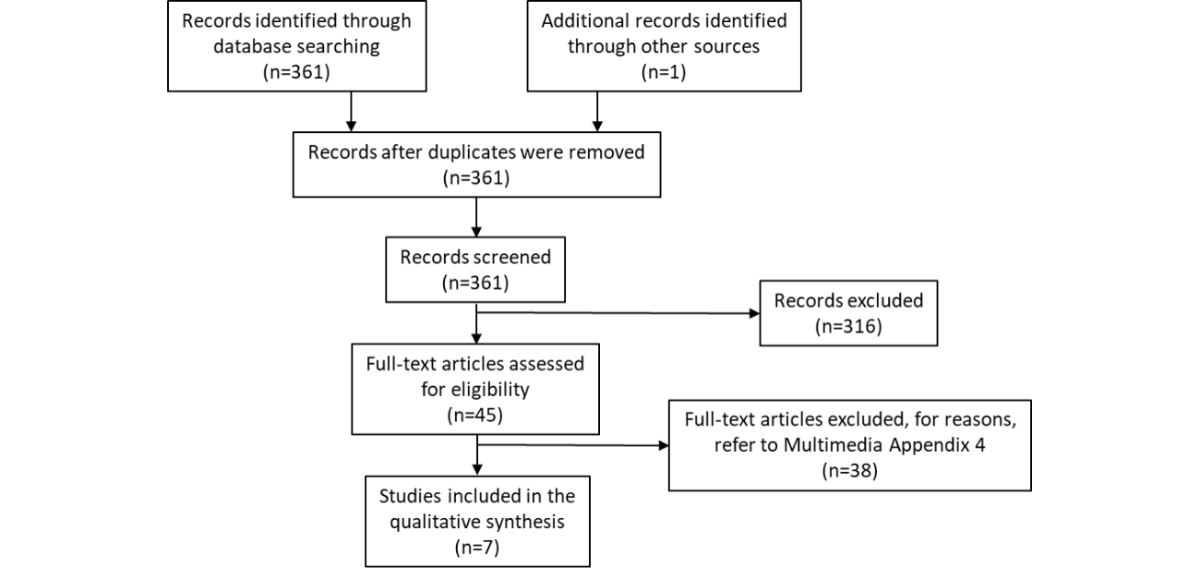
Flow of studies through the selection process.

The search retrieved 361 citations, of which 45 (12.5%) were considered potentially relevant and 7 (2%) met the inclusion criteria (Figure 1). Tables 1 and 2 provide summaries of the included reviews. The reviews varied somewhat in their objectives (eg, investigate mechanical ventilation, ventilation rates, airflow patterns, effects of humidity, or stability of bioaerosols containing coronaviruses), agents (eg, coronaviruses or influenza viruses), and settings (eg, built environment, health care settings, or public ground transportation). The reviews were published between 2007 and 2021 (median year 2020) and included a total of 47 unique virus studies published between 1961 and 2020 (median year 2005) that were relevant to our research question (median 4 studies per review including shared references; Table 3; Multimedia Appendix 3 [13,19-71]). The reasons for excluding studies at the full-text stage were documented (Multimedia Appendix 4 [7-9,11,12,24,72-103]).

**Table 1:** Summary of the characteristics of relevant reviews.

Author, year, country, agent, and setting	Purpose or objectives	Search: databases and years	Inclusion criteria	Exclusion criteria	Study designs
Author and year: Li et al [13], 2007 Country: China Agent: airborne infectious diseases Setting: multiple built environments	“1) Is there sufficient evidence to support that the ventilation rate and/or the airflow pattern are contributing cause(s) for the spread of airborne infectious diseases? 2) If so, is there good evidence/ data to support the specification and quantification of minimum ventilation requirements to minimize the transmission of airborne infectious diseases in different settings (nosocomial or otherwise)?”	MEDLINE, ISI^a^ Web of Knowledge, and ScienceDirect (1960 to March 2005); reviewed the references of retrieved articles	“Relevance of the article to the two key research questions” “Research techniques employed must have been scientifically robust, repeatable and reliable” “Original articles in English”	Conference papers and abstracts “Descriptive articles without an explicit detailed analytic component” Work before 1960	Epidemiological studies (+/− detailed ventilation studies), case-control, cohort, intervention, questionnaire, animal, mathematical modeling
Author and year: Luongo et al [19], 2016 Country: United States Agent: infectious agents Setting: buildings	To review epidemiological studies examining the association between ventilation (at least one HVAC^b^ parameter) and airborne transmission of infectious agents in buildings “To assess the quality and quantity of available data and to identify research needs”	Science Direct, Web of Knowledge, MEDLINE or PubMed, Engineering Village, and Google Scholar (search dates not reported)	“Specifically used an epidemiologic study design and that described or measured some HVAC parameter within the context of the hypothesized associations”	Modeling studies	“Epidemiologic studies investigating the association of at least one HVAC-related parameter with an infectious disease-related outcome in buildings (almost all studies reported ventilation rates or CO_2_)”
Author and year: Derby et al [20], 2017 Country: United States Agent: multiple infectious agents Setting: laboratory and multiple built environments	“To conduct a broad survey of post-1985 literature regarding the effects of low humidity on comfort, health, and IEQ [indoor environmental quality]” “To identify existing knowledge and knowledge gaps, as well as confounding variables”	Engineering Index (Compendex), Web of Science, and Google Scholar; citation search of key papers in Scopus and Google Scholar (search dates not reported); citation checking of relevant review papers	Controlled studies that focus on healthy, human participants in residences and workplaces with at least one data point where the relative humidity is 40% and provide new data and report temperature	Publication after 1985 (unless papers present unique data not previously reviewed) Review papers not analyzed in depth	Experimental studies (laboratory testing studies), transmission studies with animal models, modeling studies, and epidemiological studies
Author and year: Chirico et al [21], 2020 Country: Italy (corresponding author) Agent: SARS-CoV-1^c^, MERS-CoV^d^, or SARS-CoV-2 Setting: indoor environments	“To evaluate the COVID-19 risk associated with the presence of air-conditioning systems”	PubMed or MEDLINE, PubMed Central, Google Scholar, and medRxiv (July 11, 2020); cross-referencing	Original studies (observational and experimental) of humans in indoor environments, exposed to air-conditioning systems, with respiratory infection outbreaks caused by SARS-CoV-1, MERS-CoV, or SARS-CoV-2 Studies in English Studies with no time limit	Narrative reviews, opinions, and commentaries Experimental studies on airborne transmission of coronaviruses not associated with outbreaks	Observational and experimental studies (including modeling and CFD^e^ simulation studies)
Author and year: Zhen et al [23], 2020 Country: South Africa (corresponding author) Agent: viruses such as influenza, SARS-CoV, or MERS-CoV Setting: public ground transportation	“To assess the abilities of different interventions to decrease the incidence of droplet-based infections among people using public ground transport”	MEDLINE (PubMed), CENTRAL (Cochrane Library), Web of Science (Clarivate Analytics); reference lists of relevant reviews; WHO^f^’s database “Global Research on Coronavirus Disease (COVID-19)”	Interventions (eg, PPE^g^) and relationship to infections from viruses (eg, influenza, SARS-CoV or MERS-CoV) in “humans using public transportation (taxis, buses, trains and subways)” Studies published between 2000 and 2020 in English	“Participants/context of the intervention were healthcare workers in healthcare facilities”	Systematic reviews, clinical trials, comparative observational studies, and modeling studies (owing to limited relevant research, the authors discuss international and national guidance documents)
Author and year: da Silva et al [25], 2021 Country: Portugal Agent: SARS-CoV, MERS-CoV, and SARS-CoV-2 Setting: indoor and outdoor environments	To discuss “the viability/stability of aerosols containing SARS-CoV and MERS-CoV viruses...to provide information on potential mitigation strategies for SARS-CoV-2 airborne transmission”	PubMed or MEDLINE, Web of Science, and Scopus; references of studies were screened	Studies published since 2002 (the emergence of SARS-CoV) The virus studied was SARS- CoV, MERS-CoV, or SARS-CoV-2 Viability of the virus sampled from air was assessed Studies with no language limits	N/A^h^	Real-world sampling and laboratory studies
Author and year: Noorimotlagh et al [29], 2021 Country: Iran Agent: HCoVs^i^ Setting: laboratory experimental setups	“To collect all available studies concerning inactivation methods, environmental survival, and control and prevention strategies”	Scopus, ISI Web Science, Google Scholar, PubMed (MEDLINE), WHO, and American Centers for Disease Control and Prevention; 1990-2020	Original studies Studies published in English Studies available electronically (online) Studies that focus on disinfections, environmental survival, and control and prevention strategies of HCoVs	Review articles Book review Guidelines Book chapters Duplicate articles Short communications Conference documents Oral presentation Comments	Original research (study designs were not described, and mostly experimental laboratory-based studies appear)

^a^ISI: Institute for Scientific Information.

^b^HVAC: heating, ventilation, and air-conditioning.

^c^SARS-CoV-1: severe acute respiratory syndrome coronavirus 1.

^d^MERS-CoV: Middle East respiratory syndrome coronavirus.

^e^CFD: computational fluid dynamics.

^f^WHO: World Health Organization.

^g^PPE: personal protective equipment.

^h^N/A: not applicable.

^i^HCoV: human coronavirus.

**Table 2:** Summary of the results and conclusions from relevant reviews.

Author and year	Results	Conclusions
Li et al [13], 2007	Based on multidisciplinary consensus panel: “of the 40 studies, 18 were considered as nonconclusive or not meeting evidentiary threshold to support a direct contributory role of ventilation rate/airflow pattern to the airborne spread of infectious agents, 12 were partly conclusive or met threshold somewhat, 10 were deemed clearly conclusive supporting a direct contribution.”	“There is insufficient data to specify and quantify the minimum ventilation requirements in hospitals, schools, offices, homes and isolation rooms in relation to spread of infectious diseases via the airborne route.” “There is strong and sufficient evidence to demonstrate the association between ventilation, air movements in buildings and the transmission/spread of infectious diseases such as measles, tuberculosis, chicken pox, influenza, smallpox and SARS.”
Luongo et al [19], 2016	Of 13 studies (1988-2013), 11 were observational and 2 were intervention studies. Building-related factors (eg, ventilation rates) were associated with increased measures of illness in 11 studies. One study showed no association and one was inconclusive.	“Studies to date show an association between increased infectious illness and decreased ventilation rate, however, there are insufficient data to quantify how mechanical ventilation may affect the airborne transmission of infectious agents.” “The weight of the data implies that HVAC system factors in buildings have a role in APT; however, more studies need to be completed, with the eventual goal of a meta-analysis to integrate results.”
Derby et al [20], 2017	Approximately 70 articles were included overall. Nine papers examined the effects of humidity on viability or the transmission of airborne viruses. Four studies showed decreased virus viability at midrange (~50%) RH^a^. Five studies showed “a canonical dip between 40 and 80% RH.” Three studies suggested greater transmission at lower humidity (eg, 20%-35% vs 50% RH). One study showed the importance of ventilation rates in removing airborne viruses, especially in smaller droplets.	Influenza virus survival dips between 40% and 80% RH. “Lower humidity increased virus survival for influenza.” Survival declines with increased length of exposure. “Across many low humidity studies, ventilation rates and exposure times were noted as confounding variables.”
Chirico et al [21], 2020	A total of 14 studies of outbreaks associated with air-conditioning systems, all in Far East (Asian countries), were included. In total, 6 of 7 studies on SARS-CoV-1^b^ indirectly proved the role of HVAC. One study of MERS^c^ showed the contamination of HVAC^d^. In total, 4 of 6 studies on SARS-CoV-2 diffusion of virus through HVAC was suspected or supported by computer simulation.	There is evidence of HVAC systems facilitating the spread of coronaviruses in previous outbreaks in Asian (Far East) countries. Evidence for SARS-CoV-2 is limited and does not provide sufficient evidence that SARS-CoV-2 can be transmitted by HVAC systems. Generalization of results to other regions is limited because of the technological differences in HVAC systems.
Zhen et al [23], 2020	A total of 4 studies were included. One systematic review showed that the use of public transportation increased the risk of influenza transmission. One case-control study did not show increased risk of influenza diagnosis with the use of public transport. Two modeling studies showed that airborne infection on trains can be reduced with facemasks, adequate ventilation, and filtration in cases where nonrecirculated air is not possible.	“Filtering air being circulated within the bus can reduce airborne transmission of influenza between passengers, and improving ventilation on a train can decrease the risk of influenza infection.” Public transport increases the risk of transmission of influenza. Risk increases with trip duration and proximity to an infected individual. Modeling studies suggest that adequate ventilation could reduce transmission risk.
da Silva et al [25], 2021	A total of 11 studies were included: 8 studies on air sampling and 3 laboratory-based experimental studies. One MERS-CoV study showed decreased stability at 70% RH compared with 40% RH at 20 °C. One MERS-CoV study found high robustness and strong capability to survive (63.5% of viruses remaining infectious 60 minutes after aerosolization) at 25 °C and 79% RH. One SARS-CoV-2 study showed an aerosol survival time of 3 hours.	“Temperatures ranging from 20 °C to 25 °C and relative humidity ranging from 40% to 50% were reported to have a protective effect on viral viability for airborne SARS-CoV and MERS-CoV.” “Higher temperatures and high relative humidity can have an effect on SARS-CoV-2 viability in the environment as reported in previous studies” (conclusions relate to both indoor and outdoor environments).
Noorimotlagh et al [29], 2021	A total of 42 studies (20 of inactivation and disinfection methods, 12 of environmental survival, and 10 of prevention and control strategies) were included. One study of Phi6 showed highest virus survival at RH >85% and RH <60% with significant decrease at RH 60%-85%. At a fixed RH of 75%, infectivity decreased 2 orders of magnitude between 19 and 25 °C. One study where aerosolized MERS-CoV data were reported in da Silva et al [25]. One study of MERS-CoV found its robustness and strong capability to survive at 25 °C and 79% RH. One study showed an aerosol survival time for SARS-CoV-2 of 3 h at 40% RH and 21 to23 °C and that the stability of SARS-CoV-2 similar to SARS-CoV-1.	“Temperature and relative humidity are important factors in the survival of SARS-CoV-2.” “Disease transmission via droplets is inhibited by increasing both temperature and RH in buildings.” SARS-CoV-2 can survive in aerosols for approximately 3 hours. “Proper ventilation of the buildings in time of aerosol generating” is recommended (however, studies of ventilation were not reviewed).

^a^RH: relative humidity.

^b^SARS-COV-1: severe acute respiratory syndrome coronavirus 1.

^c^MERS: Middle East respiratory syndrome.

^d^HVAC: heating, ventilation, and air-conditioning.

Li et al [13] examined the role of ventilation (specifically, ventilation rates and airflow patterns) in the airborne transmission of infectious agents in indoor settings. The authors included 40 English-language studies overall, with 16 (40%) specific to viruses reported between 1962 and 2005 (median year 1985/1996). Of the 16 studies, 3 (19%) included multiple papers (Table 3), which increased the total count to 21. Of these 21 studies, 16 (76%) studies were epidemiological, 4 (19%) involved other observational designs, and 1 (5%) was experimental. Of the 21 studies, 3 (14%) studies had limited and 4 (19%) had no investigation of ventilation rates or airflow. Studies involved a variety of settings: hospitals, hospital wards, or health clinics (9/21, 43%); aircrafts (3/21, 14%); nursing homes (3/21, 14%); schools (2/21, 10%); high-rise apartments (2/21, 10%); an office (1/21, 5%); and an animal cage (1/21, 5%). The viral agents included severe acute respiratory syndrome coronavirus 1 (SARS-CoV-1; 7/21, 33%), influenza (5/21, 24%), measles (4/21, 19%), chicken pox (2/21, 10%), rhinovirus (1/21, 5%), common cold (1/21, 5%), and smallpox (1/21, 5%). Overall quality was assessed as good for 12 (57%) studies, average for 5 (24%) studies, and unsatisfactory for 4 (19%) studies. The researchers convened a panel of experts in medicine, public health, and engineering. They used a modified Delphi approach with a final consensus meeting to rate the “evidentiary threshold” to support their hypothesis, that is, the direct contribution of ventilation to airborne transmission. Among the virus studies, 8 (38%) were rated as conclusive, 8 (38%) were partly conclusive, and 5 (19%) were nonconclusive. Among the 8 conclusive studies, 2 (25%) examined ventilation rates and showed higher rates of infection for influenza with lower ventilation rates and 6 (75%) demonstrated an association between airflow patterns and the transmission of measles (pediatric office suite), chicken pox (hospital), smallpox (hospital), and SARS-CoV-1 (hospital). In all the studies, the bioaerosols traveled a “considerable distance,” which the reviewers noted, “seemed to be related to building design” [13] (eg, placement of heating radiator, room pressure, and functional status of return air outlet). None of the virus studies provided data to support “specification and quantification of the minimum ventilation requirements” [13].

Luongo et al [19] examined evidence from epidemiological studies for the association of mechanical ventilation (at least one HVAC parameter) with the airborne transmission of infectious agents in buildings. Although the authors included 13 English-language studies, 3 (23%) were specific to viruses; all the studies were observational and were reported between 1996 and 2011 (median year 2004). One of the studies had 2 papers, which increased the total count to 4 (Table 3). All 4 virus studies were also included in Li et al [13]. The settings included nursing homes (2/4, 50%), an office building (1/4, 25%), and a hospital (1/4, 25%). The viruses represented in the studies included influenza (2/4, 50%), SARS-CoV-1 (1/4, 25%), and rhinovirus (1/4, 25%). The review authors did not assess methodological quality but provided a narrative commentary on the strengths and limitations of each study. Of the 4 studies, 2 (50%) found an association between virus incidence rates, self-reported incidence rates, and the risk of exposure with HVAC design features. In a retrospective cohort study of a SARS-CoV-1 outbreak in a hospital, the authors measured ventilation rates and found that “proximity to index patient associated with transmission” [19]. The authors of the second study blindly adjusted outdoor air supply dampers in 3 office buildings and found a significant positive association between average CO_2_ concentration greater than 100 ppm above background and the frequency of rhinovirus detection in air filters. The third study found a lower incidence of influenza in newer nursing homes that had 100% outside air delivery (compared with older homes with 30%-70% recirculated air) and filtered room supply (compared with no filtration) during 1 season; however, data collected over 5 subsequent years, reported in the fourth study, found no clear association. None of the studies quantified the minimum ventilation requirements.

**Table 3:** Relevant studies from the included reviews that are pertinent to the overview’s research question.

	Li et al [13], 2007	Luongo et al [19], 2016	Derby et al [20], 2017	Chirico et al [21], 2020	Zhen et al [23], 2020	da Silva et al [25], 2021	Noorimotlagh et al [29], 2021	Topics
Akers et al [39], 1966			✓					Humidity
Bloch et al [40], 1985	✓							Ventilation (airflow)
Browne et al [24], 2016					✓			Ventilation (ventilation rate)
Castilla et al [41], 2013					✓			Virus survival or detection
Chen et al [42], 2011				✓				Ventilation (airflow)
de la Noue et al [43], 2014			✓					Humidity
Drinka et al [34], 1996^a,b^	✓	✓						Ventilation (airflow) and filtration
Drinka et al [44], 2002^a^	✓							Ventilation (airflow)
Drinka et al [35], 2004^a,b^	✓	✓						Ventilation (ventilation rate)
Furuya [45], 2007					✓			Ventilation (ventilation rate)
Gustafson et al [46], 1982	✓							Ventilation (airflow)
Harper [47], 1961			✓					Humidity
Hemmes et al [48], 1962			✓					Humidity
Kim et al [22], 2016				✓		✓		Ventilation (airflow and ventilation rate)
Le et al [49], 2004	✓							Ventilation (airflow)
Leclair et al [50], 1980	✓							Ventilation
Lee et al [51], 2003				✓				Virus survival or detection
Li et al [32], 2005^c^	✓			✓				Ventilation (airflow and ventilation rate)
Li et al [38], 2005	✓			✓				Ventilation (airflow)
Li et al [52], 2020				✓				Ventilation (ventilation rate)
Lowen et al [53], 2007			✓					Humidity
Lowen and Steel [54], 2014			✓					Humidity
Lu et al [55], 2020				✓				Ventilation (ventilation rate)
Mizumoto and Chowell [56], 2020				✓				Ventilation (airflow)
Moser et al [57], 1979	✓							Ventilation (ventilation rate)
Myatt et al [36], 2004	✓	✓						Ventilation (ventilation rates)
Noti et al [58], 2013			✓					Humidity
Olsen et al [59], 2003	✓							Ventilation (ventilation rate)
Prussin et al [30], 2018							✓	Humidity
Pyankov et al [27], 2018						✓	✓	Humidity
Qian et al [60], 2020				✓				Ventilation (ventilation rates)
Remington et al [61], 1985	✓							Ventilation (ventilation rate)
Riley [62], 1978^d^	✓							Ventilation (airflow)
Riley [63], 1979^d^	✓							Ventilation (airflow)
Schulman and Kilbourne [64], 1962	✓							Humidity
van Doremalen et al [26], 2013						✓	✓	Humidity
van Doremalen et al [28], 2020						✓	✓	Virus survival or detection
Wehrle et al [65], 1970	✓							Humidity
Wong et al [31], 2004^c^	✓	✓		✓				Ventilation (airflow) and humidity
Xu et al [66], 2020				✓				Ventilation
Yang and Marr [67], 2011			✓					Humidity
Yang et al [68], 2012			✓					Humidity
Yu et al [37], 2004	✓			✓				Ventilation (airflow)
Yu et al [33], 2005^c^	✓			✓				Ventilation (airflow)
Zhang et al [69], 2013				✓				Ventilation (airflow)
Zhu et al [70], 2012					✓			Ventilation (airflow)
Zitter et al [71], 2002	✓							Ventilation (airflow)
Total number of studies relevant to this overview per included review	21	4	9	14	4	4	4	N/A^e^

^a^Li et al [13] evaluated Drinka et al [34], Drinka et al [44], and Drinka et al [35] as one.

^b^Luongo et al [19] evaluated Drinka et al [34] and Drinka et al [35] as one.

^c^Li et al [13] evaluated Li et al [32], Wong et al [31], and Yu et al [33] as one.

^d^Li et al [13] evaluated Riley et al [62] and Riley et al [63] as one.

^e^N/A: not applicable.

Derby et al [20] conducted a literature review to assess the effects of low humidity (≤40% RH) on comfort, health, and indoor environmental quality. Although the review included approximately 70 papers, 9 (13%) papers examined the effects of humidity on the viability or transmission of airborne viruses. Of these 9 studies, 7 (78%) were experimental (involving laboratory testing), 1 (11%) was a reanalysis of the data from one of the experimental studies, and 1 (11%) study involved modeling. Most studies focused on influenza, with 1 study each examining Columbia SK viruses, murine norovirus, and multiple viruses (influenza, vaccinia, Venezuelan equine encephalomyelitis, and poliomyelitis). Most studies examined a wide range of RH, from approximately 5% to 25% RH at the lower range to 75% to 100% RH at the upper range. The absolute humidity was approximately ≤25 g/m^3^ in all 9 studies except 1 (11%) (which ranged from 25 to 125 g/m^3^). The review authors did not assess the methodological quality of the included studies. In terms of virus viability, 4 studies (44%) showed a reduction in midrange RH (ie, approximately 50% RH). The review authors further noted that 5 studies (56%) showed that “virus survival exhibited a canonical dip between 40 and 80% RH” [20] and that in almost all the cases, the decline in survival was correlated with increased length of exposure. In total, 3 studies (33%) examined influenza transmission. One study showed reduced influenza transmission among guinea pigs at 50% RH versus 20% to 35% RH; however, the same pattern was not found when the researchers analyzed the data based on absolute humidity. A second study examined transmission via coughing using manikins and found 5 times more infectious virus at 7% to 23% RH than at >43% RH. A modeling study of influenza virus transmission via coughing showed that the infectious virus concentration was 2.4 times more at 10% RH than at 90% RH after 10 minutes, and the ratio increased over time. They also demonstrated that the effect of humidity is related to the particle size: the settling of larger particles and inactivation of smaller particles (<5 µm) with greater humidity. They concluded that the inactivation resulting from high RH coupled with ventilation was important to remove smaller particles.

Chirico et al [21] conducted a rapid review (streamlined systematic review methods) to examine the potential role of air-conditioning (HVAC) systems in “outbreaks of coronaviruses (SARS-CoV-1, MERS-CoV, SARS-CoV-2) in indoor environments” [21]. The authors identified 14 studies published between 2003 and 2020 (n=11, 79% peer-reviewed studies and n=3, 21.4% preprints all concerning SARS-CoV-2); the studies investigated outbreaks in Hong Kong (n=7, 50%), South Korea (n=1, 7%), Japan (n=3, 21%), and China (n=3, 21%). Of 14 studies, 7 studies (50%) examined 2 outbreaks associated with SARS-CoV-1: 5 (71%) studies examined outbreaks (different areas or groups of individuals) within the same hospital, and 2 (29%) studies investigated an outbreak in the same private high-rise housing estate. Of the 7 SARS-CoV-1 studies from Chirico et al [21], 5 (71%) are shared references with Li et al [13] (Table 3). The review authors indicated that 6 of 7 SARS-CoV-1 (86%) studies indirectly demonstrated a role for the HVAC system (through epidemiological data, spatiotemporal patterns of infection, or modeling). Of 14 studies, 1 (7%) study investigated an outbreak of Middle East respiratory syndrome coronavirus (MERS-CoV) in a hospital setting and demonstrated the contamination of the HVAC system through environmental sampling [22]. Of 14 studies, 6 (43%) studies investigated outbreaks of SARS-CoV-2: 1 (17%) study examined 318 outbreaks in 120 cities in China, including community and workplace settings; 3 (50%) studies examined an outbreak on a ship in Japan; and 2 (33%) studies examined the same outbreak in a restaurant. A total of 3 (50%) observational studies suspected a role for the HVAC system, 2 (23%) studies (both of ship outbreak) did not find evidence of a role for HVAC based on the spatiotemporal distribution of cases, and 1 (17%) study (of restaurant outbreak) supported a role for HVAC by computer simulation. The review authors indicated that they were not able to appropriately evaluate the quality of the included studies. The review authors concluded that there is sufficient evidence from SARS-CoV-1 and MERS-CoV studies demonstrating a role for HVAC in the airborne transmission of the viruses; however, there was not sufficient evidence that HVAC systems play an important role in the case of SARS-CoV-2. Although there is a lack of evidence for SARS-CoV-2, there was no evidence of no role.

Zhen et al [23] conducted a rapid review of “the role of public ground transport in COVID-19 transmission” and “interventions that may reduce transmission” [23]. The authors searched for studies published since 2000 and identified 4 relevant studies, published between 2007 and 2016, namely 1 (25%) systematic review, 1 (25%) case-control study, and 2 (50%) modeling studies. The systematic review by Browne et al [24] identified 41 studies examining the risk of transmission of Influenza A (H1N1/09) (n=29, 71% studies), SARS-CoV (n=5, 12% studies), both influenza and SARS-CoV (n=2, 5% studies), MERS-CoV (n=2, 5% studies), or unspecified viruses (n=3, 7% studies) related to sea (n=6, 15% studies), ground (n=6, 15% studies), or air (n=29, 71% studies) transport. Zhen et al [23] summarized results from 4 quantitative studies included in Browne et al [24] and concluded that the “use of public transport increased the risk of influenza transmission” [23]. Zhen et al [23] identified a multicenter case-control study that showed a lower probability of Influenza A (H1N1/09) diagnosis with public transport use (metro, bus, tram, or local train) and no association with diagnosis and the use of trains, airplanes, or taxis. The case-control study was assessed as having a moderate risk of bias by Zhen et al [23]; the risk of bias was not reported for the other 3 studies (75%). Zhen et al [23] also identified 2 modeling studies: one estimated the reproduction number for influenza infection in a train, and the other tested simulations to predict influenza infection probability for 4 bus ventilation systems. The first modeling study showed that masks could decrease the reproduction number, resulting in a lower risk of disease transmission, with high-efficiency particulate air masks being more effective than surgical masks. Furthermore, doubling the ventilation rate reduced the risk, similar to the use of high-efficiency particulate air masks, and was considered more feasible and cost-effective. The second modeling study showed that influenza transmission risk can be reduced when the infected passenger is positioned closer to the exhaust opening and with high-efficiency filtration in the case where nonrecirculated air cannot be provided. Given the limited number of research studies, Zhen et al [23] also identified and discussed national and international guidance documents, for example, those published by WHO [104-110]. Although general recommendations have been made to reduce risk (eg, minimizing the use of public transport, environmental controls, respiratory etiquette, hand hygiene, and mask use), there is no indication of the empirical evidence specific to these measures, in particular mechanical ventilation.

da Silva et al [25] conducted a systematic review to discuss “the viability/stability of aerosols containing SARS-CoV and MERS-CoV viruses” with an intent “to provide information on potential mitigation strategies for SARS-CoV-2 airborne transmission” [25]. The review authors identified 11 studies. Of these 11 studies, 8 (73%) studies examined the viability of coronaviruses in air samples, but the review authors did not describe the relationship with HVAC features, including 1 (13%) MERS-CoV study [22], which was described earlier by Chirico et al [21]. The remaining 3 (27%) studies were laboratory-based experimental studies of coronaviruses. In one MERS-CoV study, the virus was aerosolized at 20 °C with 40% or 70% RH, showing decreased stability at 70% RH compared with 40% RH [26]. The other MERS-CoV study examined virus inactivation under 2 conditions [27]: common office environment (25 °C and 79% RH) and Middle Eastern region climate (38 °C and 24% RH). In the simulated office environment, “the virus demonstrated high robustness and strong capability to survive with about 63.5% of viruses remaining infectious 60 min after aerosolisation. Virus decay was much stronger for hot and dry air scenario with only 4.7% survival over 60 min procedure” [25]. One of the studies showed an aerosol survival time of 3 hours for SARS-CoV-2 [28]. The review authors did not assess the methodological quality of the included studies; however, they commented on some limitations. The review authors concluded that “higher temperatures and high relative humidity can have an effect on SARS-CoV-2 viability in the environment as reported in previous studies to this date” [25]. However, their conclusions were based on studies of both indoor and outdoor environments.

Noorimotlagh et al [29] performed a systematic review of SARS-CoV-2 literature “to collect all available studies concerning inactivation methods, environmental survival, and control and prevention strategies” [29]. Although 42 studies were identified, 4 provided information on temperature and humidity in the built environment, investigating MERS-CoV (n=2, 50%), SARS-CoV-1 and SARS-CoV-2 (n=1, 25%), and Phi6 (n=1, 25%), which is a bacteriophage used as a surrogate for viruses. All of them were laboratory-based experimental studies. The review authors did not assess or comment on the methodological quality of the included studies. The aerosolized MERS-CoV data from van Doremalen et al [26] were reported by da Silva et al [25] although not extracted by Noorimotlagh et al [29]. A MERS-CoV study found that the virus had robustness and strong capability to survive at 25 °C and 79% RH [27]; this was a shared reference with da Silva et al [25]. Although da Silva et al [25] reported the SARS-CoV-2 survival time from van Doremalen et al [28], Noorimotlagh et al [29] further clarified that the aerosol survival time of 3 hours for SARS-CoV-2 was at 40% RH and 21–23 °C and that the stability of SARS-CoV-2 was similar to that of SARS-CoV-1 [28]. The Phi6 study showed the highest virus survival at >85% RH and <60% RH with a significant decrease between 60% and 85% RH [30]. At a fixed humidity of 75% RH, infectivity decreased by 2 orders of magnitude between 19 and 25 °C [30]. The review authors concluded that “temperature and relative humidity are important factors in the survival of SARS-CoV-2” [29] and that “disease transmission via droplets is inhibited by increasing both temperature and RH in buildings” [29]. A review recommendation was “proper ventilation of the buildings in time of aerosol generating” [29]; however, studies of ventilation were not reviewed.

### Network of Included Reviews

**Figure 2:**
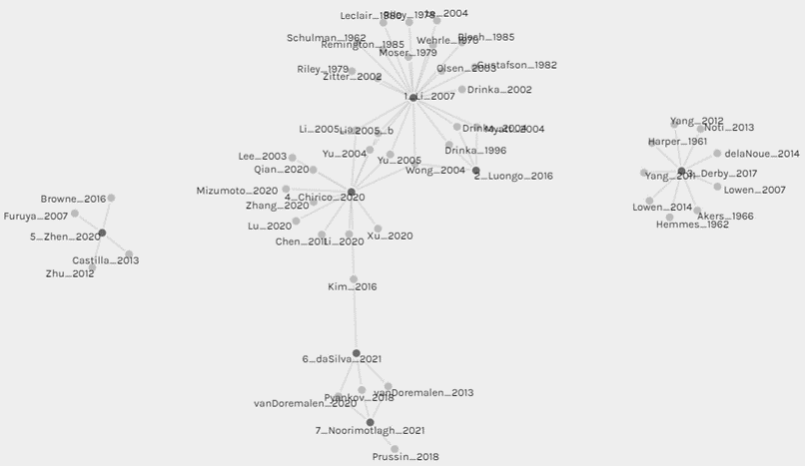
Network representing relevant references (gray circles) from the 7 included reviews (black circles): 1_Li_2007; 2_Luongo_2016; 3_Derby_2017; 4_Chirico_2020; 5_Zhen_2020, 6_daSilva_2021; 7_Noorimotlagh_2021. Shared References are as follows: 1_Li_2007, 2_Luongo_2016, and 4_Chirico_2020 share Wong_2004; 1_Li_2007 and 2_Luongo_2016 share Drinka_1996, Drinka 2004, and Myatt 2004; 1_Li_2007 and 4_Chirico_2020 share Li_2005_a, Li_2005_b, Yu_2004, and Yu_2005; 4_Chirico_2020 and 6_daSilva_2021 share Kim_2016; 6_daSilva_2021 and 7_Noorimotlagh_2021 share vanDoremalen_2013, Pyankov_2018, and vanDoremalen_2020.

The network of the 7 included reviews and their 47 references relevant to this overview was created using Palladio (Figure 2). Overall, 12 references were shared among the 7 included reviews. However, the network clearly demonstrates that Derby et al [20] and Zhen et al [23] shared no references with the 5 other reviews. In actuality, the 12 references were shared between 5 reviews (Figure 2). da Silva et al [25] and Noorimotlagh et al [29] shared 3 references regarding experimental studies of MERS-CoV and SARS-CoV-2 [26-28] (Table 3). In addition, da Silva et al [110] shared 1 reference on MERS-CoV isolation wards [22] with Chirico et al [21]. Three reviews, Li et al [13], Luongo et al [19], and Chirico et al [21], shared a reference on SARS-CoV-1 in hospital wards [31]. Li et al [13] and Chirico et al [21] shared other related references on SARS-CoV-1 in hospital wards [32,33]. Similarly, Li et al [13] and Luongo et al [19] shared studies on influenza in nursing homes [34,35] and rhinovirus in offices [36]. Li et al [13] and Chirico et al [21] shared 2 other references regarding SARS-CoV-1 in high-rise apartment complexes [37,38]. Not only were the 12 references shared only between 5 reviews, 8 of these references were shared with 1 2007 review by Li et al [13], and the remaining 4 references were shared with 1 2021 review by da Silva et al [25] (Figure 2). Although 35 of the 47 references were not shared, the 12 shared references were captured by the earliest review (2007; [13]) and one of the latest reviews (2021; [25]).

### Quality Assessment

Table 4 provides the assessments of the methodological quality of the reviews based on AMSTAR2. In total, 3 (43%) papers described themselves as systematic reviews, 2 (29%) were rapid reviews, 1 (14%) was a broad literature survey, and 1 (14%) was described simply as a review. The majority provided detailed research questions, explained study designs considered for inclusion, used a comprehensive search strategy, described the included studies, discussed the heterogeneity of results, and reported potential conflicts of interest. None or few reviews provided an a priori protocol, performed study selection and data extraction in duplicate, provided a list of excluded studies, conducted risk of bias assessments of individual studies, or reported on the sources of funding for the included studies. None of the reviews conducted a meta-analysis; all of them provided a narrative synthesis of the results and observations across the included studies. A previous review [19] spoke about the need for more well-designed studies (including representative sampling and clear and consistent measurement methods and reporting of data) with the goal of using meta-analysis to integrate the results.

**Table 4:** Methodological quality of the relevant reviews based on AMSTAR2.

AMSTAR2 question	Li et al [13], 2007	Luongo et al [19], 2016	Derby et al [20], 2017	Chirico et al [21], 2020	Zhen et al [23], 2020	da Silva et al [25], 2021	Noorimotlagh et al [29], 2021
1. Did the research questions and inclusion criteria for the review include the components of PICO?	Yes	Yes	Yes	Yes	Yes	Yes	Yes
2. Did the report of the review contain an explicit statement that the review methods were established prior to the conduct of the review and did the report justify any significant deviations from the protocol?	No	No	No	No	No	No	No
3. Did the review authors explain their selection of the study designs for inclusion in the review?	Yes	Yes	Yes	Yes	Yes	No	No
4. Did the review authors use a comprehensive literature search strategy?	Yes	Partial Yes	Partial Yes	Yes	Yes	Yes	Partial Yes
5. Did the review authors perform study selection in duplicate?	Yes	No	No	No	No	Yes	Yes
6. Did the review authors perform data extraction in duplicate?	No	No	No	No	No	No	No
7. Did the review authors provide a list of excluded studies and justify the exclusions?	No	No	No	No	No	No	No
8. Did the review authors describe the included studies in adequate detail?	Yes	Yes	Partial Yes	Yes	Yes	Yes	Yes
9. Did the review authors use a satisfactory technique for assessing the risk of bias (RoB) in individual studies that were included in the review?	No	No	No	No	Partial Yes	No	No
10. Did the review authors report on the sources of funding for the studies included in the review?	No	No	No	No	No	No	No
11. If meta-analysis was performed did the review authors use appropriate methods for statistical combination of results?	No meta-analysis conducted	No meta-analysis conducted	No meta-analysis conducted	No meta-analysis conducted	No meta-analysis conducted	No meta-analysis conducted	No meta-analysis conducted
12. If meta-analysis was performed, did the review authors assess the potential impact of RoB in individual studies on the results of the meta-analysis or other evidence synthesis?	No meta-analysis conducted	No meta-analysis conducted	No meta-analysis conducted	No meta-analysis conducted	No meta-analysis conducted	No meta-analysis conducted	No meta-analysis conducted
13. Did the review authors account for RoB in individual studies when interpreting/discussing the results of the review?	Yes	Yes	No	No	Yes	Yes	No
14. Did the review authors provide a satisfactory explanation for, and discussion of, any heterogeneity observed in the results of the review?	Yes	Yes	Yes	Yes	Yes	Yes	No
15. If they performed quantitative synthesis did the review authors carry out an adequate investigation of publication bias (small study bias) and discuss its likely impact on the results of the review?	No meta-analysis conducted	No meta-analysis conducted	No meta-analysis conducted	No meta-analysis conducted	No meta-analysis conducted	No meta-analysis conducted	No meta-analysis conducted
16. Did the review authors report any potential sources of conflict of interest, including any funding they received for conducting the review?	Yes	Yes	Yes	Yes	Yes	Yes	Yes

## Discussion

### Principal Findings

This comprehensive overview of reviews provides a map of the existing synthesized evidence on the role of HVAC in airborne virus transmission. The earliest review by Li et al [13] published in 2007 found evidence of an association between ventilation rates and airflow patterns in buildings and the transmission of viral diseases. Li et al [13] found no studies that provided minimum ventilation requirements to prevent the spread of viral diseases; however, they found 1 study showing that tuberculin conversion was significantly associated with ventilation rates of <2 air changes per hour in general patient rooms [111]. Published in 2007 shortly after the 2003 SARS-CoV-1 epidemic, Li et al [13] called for a “multidisciplinary research culture” to study outbreaks, as well as smaller-scale transmission occurrences, for filling the gap with respect to quantifying minimum ventilation standards in both clinical and nonclinical settings. A subsequent review by Luongo et al [19] published almost 10 years later, in 2016, included a subset of 4 of the virus studies identified by Li et al [13], with similar conclusions about the possible association between ventilation features (low outdoor air supply and imbalance in supply and exhaust airflow rates) and airborne virus transmission. Luongo et al [19] also pointed out the lack of data to quantify how mechanical ventilation may affect airborne transmission and the need for more well-designed multidisciplinary epidemiological studies. More recently, in response to the current COVID-19 pandemic, Chirico et al [21] examined HVAC systems and their role in the airborne transmission of coronaviruses; they concluded that there was sufficient evidence demonstrating an association for SARS-CoV-1 and MERS-CoV, whereas there was a lack of evidence for SARS-CoV-2. Derby et al [20] specifically examined the role of humidity in relation to indoor air quality; the evidence they identified was specific to influenza and showed that virus survival was lowest between 40% and 80% RH and that survival time decreased with the length of exposure to humidity. One of the studies from Noorimotlagh et al [29] indicated that aerosolized SARS-CoV-2 can survive for 3 hours at 40% RH and 21 to 23 °C [28]. In another recent review published in 2021, da Silva et al [25] examined mitigation strategies and found 2 studies demonstrating that coronavirus transmission decreased with increasing both temperature and RH in buildings. A recent review (2020) by Zhen et al [23] examined interventions to reduce virus transmission in public ground transportation; 2 modeling studies showed ventilation and filtration to be effective.

### Comparison With Prior Work

Although there is an extensive body of literature examining HVAC and its role in airborne virus transmission, there is a lack of empirical evidence to quantify the minimum standards for HVAC design features in the built environment. Previous reviews have discussed this gap, stressed the need for methodologically rigorous epidemiological studies involving multiple disciplines (eg, engineering, medicine, epidemiology, and public health), and discussed considerations for future research, including the specificity of the virus, its construction and envelope composition, the infectious dose, and the size of the particle containing the virus. The review authors have called for standardizing experimental conditions, measurements, terminologies, and reporting as well as simulating real-world conditions [19,20,25]. An important consideration in designing rigorous studies is controlling for confounding factors. HVAC systems operate in a complex environment; for example, Derby et al [20] noted several confounding variables to be considered when interpreting their findings on humidity and temperature including “variation in air exchange rate, length of organism exposure, variation in the biological structure and routes of entry, variation of pathogen survival on different fomites, and variances in human host response” [20]. They further noted that the number and complexity of the variables to consider “greatly increases the test matrices required” [20] to build a comprehensive evidence base. Studies have also demonstrated the importance of the positioning of the infected person relative to HVAC features and other occupants, mobility patterns and activities (eg, type and intensity of respiratory activity) of the occupants, time spent within a space, occupancy, and occupant density. Despite the specification of airflow parameters, the flow of air in occupied spaces is almost always turbulent (vs laminar) such that particles “are constantly mixing and moving in varied ways across a space,” making assessments and predictions challenging [112]. Finally, research results need to be interpreted in light of the technological differences in the HVAC systems around the world [21]. Engineers have developed sophisticated methods (through modeling, computational fluid dynamics, etc) that allow for the isolation of features and control for confounding variables. However, these studies rely on many assumptions that may not hold in real-world settings or are specific to an assumed building design or configuration. In addition, these studies may isolate 1 component in the chain of transmission, which does not necessarily equate to the actual disease (eg, the detection of viral particles vs infectivity vs disease outcomes) [19,20,25]. The results from modeling studies need to be considered alongside epidemiological studies. Previous reviews have highlighted many challenges with studying outbreaks: Li et al [13] mentioned that the “most inherent limitation in almost all existing investigations is due to the rapid disappearance of airborne evidence of infection, once the infectious period is over” [13]. They proposed as a solution “contemporaneous air-sampling and environmental measurements” [13] in locations during a patient’s illness, which could be extended to locations of high use or occupancy during a pandemic or seasonal epidemics.

### Strengths and Limitations

The strengths of this study include its comprehensiveness and the use of methods to avoid bias, such as the prespecification of inclusion and exclusion criteria and involvement of at least two reviewers at all stages. The main limitation stems from the limits of the included reviews. We initially intended to include only systematic reviews that met internationally recognized definitions and methodological expectations. However, we relaxed our criteria given that many reviews did not meet this standard. Although most reviews prespecified their research question and conducted a comprehensive search, few conducted study selection and data extraction in duplicate as recommended to avoid bias, and very few assessed the methodological quality or risk of bias of the included studies, which is key to determining the validity and certainty of the available evidence. We also did not find reviews of all HVAC design features; for example, none of the included reviews examined UV germicidal irradiation (although a recent narrative review has been published in the context of COVID-19 [72]), and only a small number of studies across the reviews examined filtration.

### Implications

The findings of this overview have several implications for public health measures to mitigate the spread of viral transmission in buildings. First, ventilation rates and airflow patterns have been shown to be associated with virus transmission. Second, humidity and temperature are associated with virus survival. Third, filtration can be effective in removing pathogens if the filter rating is commensurate with the size of the particles of interest [19]. The reviews have also mentioned the importance of regular maintenance of HVAC systems and features to ensure optimal functioning. Across the reviews, there was a clearly stated need for more methodologically rigorous interdisciplinary research with a specific focus on quantifying the minimum specifications for HVAC features. Although one of the reviews did not find sufficient evidence of association between HVAC and airborne transmission specific to SARS-CoV-2, the authors did advise (based on evidence for MERS-CoV and SARS-CoV-1) that attention be given to the design and management of HVAC systems as a precautionary measure until further evidence indicates otherwise [21].

### Conclusions

Airborne transmission is now recognized as a route of transmission for different viruses, including coronaviruses, specifically SARS-CoV-2, which has been the source of immense global impacts in terms of morbidity, mortality, and the peripheral effects of pandemic restrictions. HVAC systems and their specific features have the potential to mitigate transmission in built environments: there is evidence that ventilation rates, airflow patterns, humidity, temperature, and filtration can influence virus transmission. Enhancing HVAC systems in built environments (including schools, office buildings, commercial spaces, recreation centers, and transport vehicles) could have important implications for the current pandemic as well as seasonal epidemics and other diseases and impacts that are associated with general indoor air quality. These measures will be of utmost relevance to countries that experience cooler climates and where people spend an inordinate amount of time (80%-90%) indoors. Moreover, mitigation strategies that do not rely on human behavior and result in other (eg, social) consequences will be more sustainable [21]. This overview synthesized 7 previous reviews that included 47 studies examining HVAC design features and their effects on the airborne transmission of viruses, serving as a starting point for future systematic reviews and identifying priorities for primary research.

## Appendix

Multimedia Appendix 1 Search strategies for Ovid MEDLINE and Compendex.

Multimedia Appendix 2 Inclusion and exclusion criteria for the overview of reviews [16].

Multimedia Appendix 3 Relevant studies from the included reviews that are pertinent to the overview’s research question (with full citations).

Multimedia Appendix 4 Publications excluded at full-text screening with reasons.

## Abbreviations

HVAC: heating, ventilation, and air-conditioning
MERS-CoV: Middle East respiratory syndrome coronavirus
RH: relative humidity
SARS-CoV-1: severe acute respiratory syndrome coronavirus 1
WHO: World Health Organization
