# Supplementary material for: The Impact of Heating, Ventilation, and Air-Conditioning Design Features on the Transmission of Viruses, Including SARS-CoV-2: Overview of Reviews
Source: Interact J Med Res. 2022 Dec 23;11(2):e37232. doi: 10.2196/37232 (PMC9823592; doi:10.2196/37232)
Supplement: Multimedia Appendix 1 [file ijmr_v11i2e37232_app1.pdf]

Multimedia Appendix 1. Search Strategies for Ovid MEDLINE and Compendex

Database: Ovid MEDLINE(R) ALL 1946 to Present

Search Strategy:

#	Searches
1	exp Aerosols/
2	Air Microbiology/
3	exp Viruses/
4	(aerosol or aerosols or bioaerosol or bioaerosols).mp.
5	droplet nuclei.mp.
6	infectio*.mp.
7	(pathogen or pathogens).mp.
8	(virus or viruses or viral or virome).mp.
9	or/1-8 [MeSH + Keywords – Virus concept]
10	Air Conditioning/
11	Air Filters/ or Filtration/
12	Humidity/
13	Ventilation/
14	Ultraviolet Rays/
15	air condition*.mp.
16	(air change rate or air change rates or air changes per hour or air exchange rate or air exchange rates or air exchanges per hour).mp.
17	(airflow or air flow).mp.
18	built environment.mp.
19	computational fluid dynamics.mp.
20	((distance adj6 index) or long distances).mp.
21	HVAC.mp.
22	(filter or filters or filtration).mp.
23	humidity.mp.
24	(ultraviolet or UV).mp.
25	ventilat*.mp.
26	or/10-25 [MeSH + Keywords – HVAC concept]
27	Air Pollution, Indoor/
28	exp Disease Transmission, Infectious/
29	(indoor adj1 (air quality or environment*)).mp.
30	transmission.mp.
31	or/27-30 [MeSH + Keywords – Transmission concept]
32	9 and 26 and 31
33	remove duplicates from 32
34	(pubmed or medline or cochrane or scopus or cinahl).tw. or ((systematic* or evidence-based or scoping or umbrella) adj3 (review* or overview*)).pt,mp,jw. or meta-analy*.pt,mp. or (meta-analy* or metaanalys* or research-synthesis).tw. or search*.ab. or (hta or technology assessment).mp,jw.
35	33 and 34
36	remove duplicates from 35

MeSH = Medical Subject Headings

Database: Compendex
Query:
((((((systematic* OR evidence-based OR scoping OR umbrella AND review*OR overview*) WN ST) AND (1884-2021 WN YR)) OR (((systematic* OR evidence-based OR scoping OR umbrella AND review* OR overview*) WN KY)AND (1884-2021 WN YR)) OR (((hta OR technology assessment) WN ST) AND(1884-2021 WN YR)) OR (((hta OR technology assessment) WN KY) AND (1884-2021 WN YR)) OR (((search*) WN AB) AND (1884-2021 WN YR)) OR (((meta-analy* OR metaanalys* OR research-synthesis) WN KY) AND (1884-2021 WN YR))OR (((pubmed OR medline OR cochrane OR scopus OR cinahl) WN KY) AND(1884-2021 WN YR))) AND (1884-2021 WN YR)) AND ((((((((Aerosols OR Viruses) WN CV)) AND (1884-2020 WN YR)) OR (((aerosol OR aerosols OR bioaerosol OR bioaerosols OR {droplet nuclei} OR infectio* OR pathogen OR pathogens OR virus OR viruses OR viral OR virome) WN KY) AND (1884-2020 WNYR))) AND (1884-2020 WN YR)) AND (((((({Air conditioning} OR {Air filters} OR Filtration OR {Atmospheric humidity} OR HVAC OR Ventilation OR {Ultraviolet radiation}) WN CV)) AND (1884-2020 WN YR)) OR (((air condition* OR {air change rate} OR {air change rates} OR {air changes per hour} OR {air exchange rate} OR {air exchange rates} OR {air exchanges per hour} OR airflow OR {airflow} OR HVAC OR filter OR filters OR filtration OR humidity OR ultraviolet ORUV OR ventilat*) WN KY) AND (1884-2020 WN YR))) AND (1884-2020 WN YR))AND (((((({Indoor air pollution} OR {Disease control}) WN CV)) AND (1884-2020WN YR)) OR ((({indoor air quality} OR indoor environment* OR transmission) WNKY) AND (1884-2020 WN YR))) AND (1884-2020 WN YR))) AND (1884-2020WN YR)))
