# Supplementary material for: The Impact of Heating, Ventilation, and Air-Conditioning Design Features on the Transmission of Viruses, Including SARS-CoV-2: Overview of Reviews
Source: Interact J Med Res. 2022 Dec 23;11(2):e37232. doi: 10.2196/37232 (PMC9823592; doi:10.2196/37232)
Supplement: Multimedia Appendix 2 [file ijmr_v11i2e37232_app2.pdf]

Multimedia Appendix 2. Inclusion and exclusion criteria for the overview of reviews [16]

Item	Inclusion criteria	Exclusion criteria
Agent	• Viruses<br>• Aerosols<br>• Bioaerosols<br>• Droplet nuclei<br>• Pathogens<br><br>*We planned a staged process: if we identify reviews specific to viruses, we will not include reviews not specific to virus.*	
HVAC / Mechanical ventilation	Mechanical ventilation or HVAC overall, or specific to one or more of the following HVAC design features:<br>• Ventilation (ventilation rate, air changes per hour, air exchange, airflow pattern, pressurization)<br>• Filtration (air filtration, filter type, MERV rating, filter age and/or use, pressure drop, holding capacity, replacement, change frequency)<br>• Ultraviolet germicidal irradiation (UVGI; power, dose, uniformity of dose, flow rate, bioaerosol inactivation efficiency, location)<br>• Humidity or relative humidity	• Transmission unrelated to ventilation<br>• Examines only natural ventilation
Setting	• Office buildings<br>• Public buildings (e.g., schools, day cares)<br>• Residential buildings<br>• Hospitals and other healthcare facilities (e.g., clinics)<br>• Transport vehicles (e.g., aircraft) or hubs (e.g., airports)	• Outdoor settings<br>• Indoor settings with natural ventilation
Outcomes	Quantitative data evaluating the correlation or association between virus transmission and mechanical ventilation or specific HVAC features	Qualitative data
Study design	Systematic review, i.e., use of “explicit, systematic methods…aimed at minimizing bias” to “identify, appraise and synthesize all the empirical evidence that meets pre-specified eligibility criteria to answer a specific research question”. (https://www.cochranelibrary.com/about/about-cochrane-reviews)<br><br>*We planned a staged process: we would include reviews that are not described as systematic if they searched at least 2 databases and/or were specific to viruses and met all other inclusion criteria.*	• Primary studies<br>• Commentaries, opinion pieces
Language	English	Non-English
Year	No restrictions	
Publication status	Published and/or peer-reviewed	Unpublished and/or not peer-reviewed

HVAC = heating, ventilation, and air conditioning; MERV = minimum efficiency reporting value; UVGI = ultraviolet germicidal irradiation
