# Supplementary material for: The Impact of Heating, Ventilation, and Air-Conditioning Design Features on the Transmission of Viruses, Including SARS-CoV-2: Overview of Reviews
Source: Interact J Med Res. 2022 Dec 23;11(2):e37232. doi: 10.2196/37232 (PMC9823592; doi:10.2196/37232)
Supplement: Multimedia Appendix 3 [file ijmr_v11i2e37232_app3.pdf]

Multimedia Appendix 3. Relevant studies from the included reviews that are pertinent to the overview's research question (with full citations)

Citation	Li et al [13], 2007	Luongo et al [19], 2016	Derby et al [20], 2017	Chirico et al [21], 2020	Zhen et al [23], 2020	da Silva et al [32], 2021	Noorimotlagh et al [36], 2021	Topic(s)
Akers T, Bond S, Goldberg L. Effect of temperature and relative humidity on survival of airborne Columbia SK group viruses. *Appl Microbiol* 1966;14:361-4.[39] https://doi.org/10.1128/am.14.3.361-364.1966			√					Humidity
Bloch AB, Orenstein WA, Ewing WM, et al. Measles outbreak in a pediatric practice: airborne transmission in an office setting. *Pediatr* 1985;75:676-83.[40]	√							Ventilation (airflow)
Browne A, St-Onge Ahmad S, Beck CR, et al. The roles of transportation and transportation hubs in the propagation of influenza and coronaviruses: A systematic review. *J Travel Med* 2016;18:1-7.[24] https://doi.org/10.1093/jtm/tav002					√			Ventilation (ventilation rate)
Castilla J, Godoy P, Domínguez Á, et al. Risk factors and effectiveness of preventive measures against influenza in the community. *Influenza Other Respir Viruses* 2013;7:177-83.[41] https://doi.org/10.1111/j.1750-2659.2012.00361.x					√			Virus survival/ detection
Chen C, Zhao B, Yang X, et al. Role of two-way airflow owing to temperature difference in severe acute respiratory syndrome transmission: revisiting the largest nosocomial severe acute respiratory syndrome outbreak in Hong Kong. *J R Soc Interface* 2011;8:699-710.[42] https://doi.org/10.1098/rsif.2010.0486				√				Ventilation (airflow)
de la Noue AC, Estienney M, Aho S, et al. Absolute humidity influences the seasonal persistence and infectivity of human norovirus. *Appl Environ Microbiol* 2014;80:7196-205.[43] https://doi.org/10.1128/AEM.01871-14			√					Humidity

Drinka PJ, Krause P, Schilling M, Miller BA, Shult P, Gravenstein S. Report of an outbreak: nursing home architecture and influenza-A attack rates. *J Am Geriatr Soc* 1996;44:910-13.[34][a,b] https://doi.org/10.1111/j.1532-5415.1996.tb01859.x	√	√						Ventilation (airflow) Filtration
Drinka PJ, Krause P, Nest L, Gravenstein S, Goodman B, Shult P. Delays in the application of out-break control prophylaxis for influenza A in a nursing home. *Infect Control Hosp Epidemiol* 2002;23:600-3.[44][a] https://doi.org/10.1086/501978	√							Ventilation (airflow)
Drinka PJ, Krause P, Nest L, Tyndall D. Report of an outbreak: nursing home architecture and influenza-A attack rates: update. *J Am Geriatr Soc* 2004;52:847-48.[35][a,b] https://doi.org/10.1111/j.1532-5415.2004.52230_6.x	√	√						Ventilation (ventilation rate)
Furuya H. Risk of transmission of airborne infection during train commute based on mathematical model. *Environ Health Prev Med* 2007;12:78-83.[45] https://doi.org/10.1265/ehpm.12.78					√			Ventilation (ventilation rate)
Gustafson TL, Lavely GB, Brawner ER Jr, Hutcheson RH Jr, Wright PF, Schaffoer W. An outbreak of airborne nosocomial varicella. *Pediatr* 1982;70:550-6.[46]	√							Ventilation (airflow)
Harper GJ. Airborne micro-organisms: survival tests with four viruses. *J Hyg* 1961;59:479-86.[47] https://doi.org/10.1017/S0022172400039176			√					Humidity
Hemmes JH, Winkler KC, Kool SM. Virus survival as a seasonal factor in influenza and poliomyelitis. *Antonie Van Leeuwenhoek* 1962;28:221-33.[48]			√					Humidity
Kim SH, Chang SY, Sung M, et al. Extensive viable Middle East respiratory syndrome (MERS) coronavirus contamination in air and surrounding environment in MERS isolation wards. *Clin Infect Dis* 2016;63:363-69.[22] https://doi.org/10.1093/cid/ciw239				√		√		Ventilation (airflow, ventilation rate)
Le DH, Bloom SA, Nguyen QH, et al. Lack of SARS transmission among public hospital workers, Vietnam. *Emerg Infect Dis* 2004;10:265-68.[49] https://doi.org/10.3201/eid1002.030707	√							Ventilation (airflow)

Leclair JM, Zaia JA, Levin MJ, Congdon RG, Goldmann DA. Airborne transmission of chickenpox in a hospital. *N Engl J Med* 1980;302:450-53.[50] https://doi.org/10.1056/NEJM198002213020807	✓							Ventilation
Lee N, Hui D, Wu A, et al. A major outbreak of severe acute respiratory syndrome in Hong Kong. *N Engl J Med* 2003;348;1986-94.[51] https://doi.org/10.1056/NEJMoa030685				✓				Virus survival/ detection
Li Y, Huang X, Yu IT, Wong TW, Qian H. Role of air distribution in SARS transmission during the largest nosocomial outbreak in Hong Kong. *Indoor Air* 2005;15:83–95.[32][c] https://doi.org/10.1111/j.1600-0668.2004.00317.x	✓			✓				Ventilation (airflow, ventilation rate)
Li Y, Duan S, Yu ITS, Wong TW. Multi-zone modeling of probable SARS virus transmission by airflow between flats in Block E, Amoy Gardens. *Indoor Air* 2005;15:96–111.[38] https://doi.org/10.1111/j.1600-0668.2004.00318.x	✓			✓				Ventilation (airflow)
Li Y, Qian H, Hang J, et al. Evidence for probable aerosol transmission of SARS-CoV-2 in a poorly ventilated restaurant. medRxiv 2020.[52] https://doi.org/10.1101/2020.04.16.20067728				✓				Ventilation (ventilation rate)
Lowen AC, Mubareka S, Steel J, Palese P. Influenza virus transmission is dependent on relative humidity and temperature. *PLoS Pathog* 2007;3:e151.[53] https://doi.org/10.1371/journal.ppat.0030151			✓					Humidity
Lowen AC, Steel J. Roles of humidity and temperature in shaping influenza seasonality. *J Virol* 2014;88:7692-5.[54] https://doi.org/10.1128/JVI.03544-13			✓					Humidity
Lu J, Gu J, Li K, et al. COVID-19 outbreak associated with air conditioning in restaurant, Guangzhou, China, 2020. *Emerg Infect Dis* 2020;26:1628-31.[55] https://doi.org/10.3201/eid2607.200764				✓				Ventilation (ventilation rate)
Mizumoto K, Chowell G. Transmission potential of the novel coronavirus (COVID-19) onboard the diamond Princess Cruises Ship, 2020. *Infect Dis Model* 2020;5:264–70.[56] https://doi.org/10.1016/j.idm.2020.02.003				✓				Ventilation (airflow)

Moser MR, Bender TR, Margolis HS, Noble GR, Kendal AP, Ritter DG. An outbreak of influenza aboard a commercial airliner. *Am J Epidemiol* 1979;110:1-6.[57] https://doi.org/10.1093/oxfordjournals.aje.a112781	✓							Ventilation (ventilation rate)
Myatt TA, Johnston SL, Zuo Z, Wand M, Kebadze T, Rudnick S, Milton DK. Detection of airborne rhinovirus and its relation to outdoor air supply in office environments. *Am J Respir Crit Care Med* 2004;169;1187-90.[36] https://doi.org/10.1164/rccm.200306-760OC	✓	✓						Ventilation (ventilation rates)
Noti JD, Blachere FM, McMillen CM, et al. Beezhold. High humidity leads to loss of infectious influenza virus from simulated coughs. *PloS One* 2013;8:e57485.[58] https://doi.org/10.1371/journal.pone.0057485			✓					Humidity
Olsen SJ, Chang HL, Cheung TY, et al. Transmission of the severe acute respiratory syndrome on aircraft. *N Engl J Med* 2003;349:2416-22.[59] https://doi.org/10.1056/NEJMoa031349	✓							Ventilation (ventilation rate)
Prussin AJ, Schwake DO, Lin K, Gallagher DL, Buttling L, Marr LC. Survival of the enveloped virus Phi6 in droplets as a function of relative humidity, absolute humidity, and temperature. *Appl Environ Microbiol* 2018;84:e00551–e00518.[30] https://doi.org/10.1128/AEM.00551-18							✓	Humidity
Pyankov OV, Bodnev SA, Pyankova OG, Agranovski IE. Survival of aerosolized coronavirus in the ambient air. *J Aerosol Sci* 2018;115:158-63.[27] https://doi.org/10.1016/j.jaerosci.2017.09.009						✓	✓	Humidity
Qian H, Miao T, Liu L, Zheng X, Luo D, Li Y. Indoor transmission of SARS-CoV-2. *medRxiv* 2020.[60] https://doi.org/10.1101/2020.04.04.20053058				✓				Ventilation (ventilation rates)
Remington PL, Hall WN, Davis IH, Herald A, Gunn RA. Airborne transmission of measles in a physician's office, *JAMA* 1985;253:1574-7.[61] https://doi.org/10.1001/jama.1985.03350350068022	✓							Ventilation (ventilation rate)
Riley RL. Airborne spread of measles in a suburban elementary school. *Am J Epidemiol* 1978;107:421-32.[62][d] https://doi.org/10.1093/oxfordjournals.aje.a112560	✓							Ventilation (airflow)

Riley RL. Indoor spread of respiratory infection by recirculation of air. *Bull Eur Physiopathol Respir* 1979;15:699–705.[63] [d]	✓							Ventilation (airflow)
Schulman JL, Kilbourne ED. Airborne transmission of influenza virus infection in mice. *Nature* 1962;195: 1129-30.[64] https://doi.org/10.1038/1951129a0	✓							Humidity
van Doremalen N, Bushmaker T, Munster VJ. Stability of Middle East respiratory syndrome coronavirus (MERS-CoV) under different environmental conditions. *Euro Surveill* 2013;18:1-4.[26] https://doi.org/10.2807/1560-7917.ES2013.18.38.20590						✓	✓	Humidity
van Doremalen N, Bushmaker T, Phil M, et al. Aerosol and surface stability of SARS-CoV-2 as compared with SARS-CoV-1. *N Engl J Med* 2020;382:1564-67.[28] https://doi.org/10.1056/NEJMc2004973						✓	✓	Virus survival/ detection
Wehrle PF, Posch J, Richter KH, Henderson DA. An airborne outbreak of smallpox in a German hospital and its significance with respect to other recent outbreaks in Europe. *Bull World Health Organ* 1970;43:669-79.[65] https://www.ncbi.nlm.nih.gov/pmc/articles/PMC2427800/	✓							Humidity
Wong TW, Li CK, Tam W, et al. Cluster of SARS among medical students exposed to single patient, Hong Kong. *Emerg Infect Dis* 2004;10:269-76.[31][c] https://doi.org/10.3201/eid1002.030452	✓	✓		✓				Ventilation (airflow); Humidity
Xu P, Qian H, Miao T, et al. Transmission routes of Covid-19 virus in the Diamond Princess Cruise ship. *medRxiv* 2020.[66] https://doi.org/10.1101/2020.04.09.20059113				✓				Ventilation
Yang W, Marr LC. Dynamics of airborne influenza A viruses indoors and dependence on humidity. *PloS One* 2011;6:e21481.[67] https://doi.org/10.1371/journal.pone.0021481			✓					Humidity
Yang W, Elankumaran S, Marr LC. Relationship between humidity and influenza A viability in droplets and implications for influenza's seasonality. *PloS One* 2012;7:e46789.[68] https://doi.org/10.1371/journal.pone.0046789			✓					Humidity

Yu IT, Li Y, Wong TW, et al. Evidence of airborne transmission of the severe acute respiratory syndrome virus. *N Engl J Med* 2004;350:1731-39.[37] https://doi.org/10.1056/NEJMoa032867	✓			✓				Ventilation (airflow)
Yu ITS, Wong TW, Chiu YL, Lee N, Li Y. Temporal-spatial analysis of severe acute respiratory syndrome among hospital inpatients. *Clin Infect Dis* 2005;40:1237-43.[33][c] https://doi.org/10.1086/428735	✓			✓				Ventilation (airflow)
Zhang l, Peng Z, Ou J, et al. Protection by face masks against influenza A(H1N1)pdm09 virus on trans-Pacific passenger aircraft, 2009. *Emerg Infect Dis* 2013;19:1403-10.[69] https://doi.org/10.3201/eid1909.121765				✓				Ventilation (airflow)
Zhu S, Srebric J, Spengler JD, Demokritou P. An advanced numerical model for the assessment of airborne transmission of influenza in bus microenvironments. *Build Environ* 2012;47(1):67-75.[70] https://doi.org/10.1016/j.buildenv.2011.05.003					✓			Ventilation (airflow)
Zitter JN, Mazonson PD, Miller DP, Hulley SB, Balmes JR. Aircraft cabin air recirculation and symptoms of the common cold. *JAMA* 2002;288:483-86.[71] https://doi.org/10.1001/jama.288.4.483	✓							Ventilation (airflow)
Total number of studies relevant to the overview per included review	21	4	9	14	4	4	4	

[a]Li et al [13] evaluated Drinka et al [34], Drinka et al [44], and Drinka et al [35] as one.
[b]Luongo et al [19] evaluated Drinka et al [34] and Drinka et al [35] as one.
[c]Li et al [13] evaluated Li et al [32], Wong et al [31], and Yu et al [33] as one.
[d]Li et al [13] evaluated Riley et al [62] and Riley et al [63] as one.
