# Supplementary material for: The Impact of Heating, Ventilation, and Air-Conditioning Design Features on the Transmission of Viruses, Including SARS-CoV-2: Overview of Reviews
Source: Interact J Med Res. 2022 Dec 23;11(2):e37232. doi: 10.2196/37232 (PMC9823592; doi:10.2196/37232)
Supplement: Multimedia Appendix 4 [file ijmr_v11i2e37232_app4.pdf]

Multimedia Appendix 4. Publications excluded at full-text screening with reasons

First author, year	Citation	Reasons for exclusion
Abd El-Wahab et al [73], 2020	Abd El-Wahab EW, Eassa SM, Metwally M, et al. SARS-CoV-2 transmission channels: a review of the literature. *MEDICC Rev* 2020;22:51-69.	Study design: not a systematic review (narrative review of transmission modes and prevention and control strategies)
Ahlawat et al [74], 2020	Ahlawat A, Wiedensohler A, Mishra SK. An overview on the role of relative humidity in airborne transmission of SARS-CoV-2 in indoor environments. *Aerosol Air Qual Res* 2020;20:1856-61.	Study design: not a systematic review (narrative review of relative humidity and airborne transmission of SARS-CoV-2 in indoor environments)
Bing et al [75], 2018	Bing-Yuan, Zhang Y-H, Leung NHL, Cowling BJ, Yang Z-F. Role of viral bioaerosols in nosocomial infections and measures for prevention and control. *J Aerosol Sci* 2018;117:200-11.	Study design: not a systematic review (narrative review of viral bioaerosols and nosocomial infections in healthcare settings)
Birgand et al [76], 2020	Birgand G, Peiffer-Smadja N, Fournier S, et al. Assessment of air contamination by SARS-CoV-2 in hospital settings. *JAMA Netw Open* 2020;3:e2033232.	HVAC: no comparison/quantitative data of HVAC design features
Brankston et al [11], 2007	Brankston G, Gitterman L, Hirji Z, Lemieux C, Gardam M. Transmission of influenza A in human beings. *Lance Infect Dis* 2007;7:257-65.	HVAC: no HVAC design features (focus on route of influenza A transmission in humans)
Browne et al [24], 2016	Browne A, St-Onge Ahmad S, Beck CR, Nguyen-Van-Tam JS. The roles of transportation and transportation hubs in the propagation of influenza and coronaviruses: a systematic review. *J Travel Med* 2016;18:1-7.	HVAC: no HVAC design features (focus on role of transportation and transportation hubs in transmission of influenza and coronaviruses)
Carter [77], 2020	Carter A. Can orthodontic care be safely delivered during the COVID-19 pandemic? Recommendations from a literature review. *Am J Orthod Dentofacial Orthop* 2020;21:66-7.	Study design: not systematic review (commentary)
Cho et al [78], 2019	Cho E-M, Hong HJ, Park SH, Yoon DK, Goung SJN, Lee CM. Distribution and influencing factors of airborne bacteria in public facilities used by pollution-sensitive population: a meta-analysis. *Inter J Environ Res Public Health* 2019;16:1483.	Study design: not a systematic review (literature review and field study with pooling of data)<br>Agent: bacteria; not specific to virus
Comber et al [7], 2020	Comber L, Murchu EO, Drummond L, et al. Airborne transmission of SARS-CoV-2 via aerosols. *Rev Med Virol* 2020;e2184.	HVAC: no HVAC design features
Edelson [79], 2012	Edelson PJ. Patterns of measles transmission among airplane travelers. *Travel Med Infect Dis* 2012;10(5-6):230-5.	HVAC: no HVAC design features
Emmerich et al [80], 2013	Emmerich SJ, Heinzerling D, Choi J, Persily AK. Multizone modeling of strategies to reduce the spread of airborne infectious agents in healthcare facilities. *Build Environ* 2013;60:105-115.	Study design: primary research (modelling/simulation study), not a systematic review
Fox et al [81], 2020	Fox GJ, Redwood L, Chang V, Ho J. The effectiveness of individual and environmental infection control measures in reducing the transmission of *Mycobacterium tuberculosis*: a systematic review. *Clin Infect Dis* 2020;72:1-12.	Agent: not specific to virus
Guo et al [82], 2019	Guo W, Cronk R, Scherer E, Oommen R, Brogan J, Sarr M, Bartram J. A systematic scoping review of environmental health conditions in penal institutions. *Int J Hyg Environ Health* 2019;222:790-803.	Agent: not specific to virus<br>HVAC: no association of virus and HVAC design features

Hertzberg et al [83], 2016	Hertzberg VS, Weiss H. On the 2-row rule for infectious disease transmission on aircraft. *Ann Glob Health* 2016;82:819-23.	Study design: not a systematic review<br>HVAC: no HVAC design features
Irwin et al [84], 2011	Irwin CK, Yoon KJ, Wang C, Hoff SJ, Zimmerman JJ, Denagamage T, O'Connor AM. Using the systematic review methodology to evaluate factors that influence the persistence of influenza virus in environmental matrices. *Appl Environ Microbiol* 2011;77:1049-60.	HVAC: examines temperature and humidity but not in context of HVAC and built environment
Khalefa et al [85], 2021	Khalefa MA, Khadabadi NA, Moores TS, et al. Evidence-based review of safe theatre practice during the COVID-19 pandemic beyond personal protective equipment. *Ann R Coll Surg Engl* 2020;103:88-95.	Study design: not systematic review
Leitmeyer et al [86], 2016	Leitmeyer K, Adlhoch C. Influenza transmission on aircraft: a systematic literature review. *Epidemiol* 2016;26:743-51.	HVAC: no HVAC design features (focus on transmission of influenza aboard aircraft)
Medical Advisory Secretariat [87], 2005	Medical Advisory Secretariat. Air cleaning technologies: an evidence-based analysis. *Ont Health Technol Assess Ser* 2005;5.	HVAC: no HVAC design features (focus on in-room air cleaners, no studies found)
Moffa et al [88], 2019	Moffa M, Cronk R, Fejfar D, Dancausse S, Padilla LA, Bartram J. A systematic scoping review of environmental health conditions and hygiene behaviors in homeless shelters. *Inter J Hyg Environ Health* 2019;222:335-46.	Agent: not specific to virus
Noorimotlagh et al [9], 2021	Noorimotlagh Z, Jaafarzadeh N, Martinez SS, Mirzaee SA. A systematic review of possible airborne transmission of the COVID-19 virus (SARS-CoV-2) in the indoor air environment. *Environ Res*. 2021;193:110612.	HVAC: no HVAC design features (focus on airborne transmission)
Perrone et al [89], 2021	Perrone G, Giuffrida M, Bellini V, et al. Operating room setup: how to improve health care professionals safety during pandemic COVID-19—A quality improvement study. *J Laparoendosc Adv Surg Tech* 2021;31:85-9.	HVAC: study examines recommendations and guidelines for operating room set-up; does not search for research about HVAC
Raeiszadeh et al [72], 2020	Raeiszadeh M, Adeli B. A critical review on ultraviolet disinfection systems against COVID-19 outbreak: applicability, validation, and safety considerations. *ACS Photonic*s 2020;7:2941-51.	Study design: not a systematic review (narrative review of ultraviolet disinfection systems against COVID-19 outbreak)
Rahimi et al [8], 2021	Rahimi NR, Fouladi-Fard R, Aali R, et al. Bidirectional association between COVID-19 and the environment: a systematic review. *Environ Res* 2021;194:110692.	HVAC: no HVAC design features (focus on route of transmission)
Ramos et al [90], 2020	Ramos CCR, Roque JLA, Sarmiento DB, Suarez LEG, Sunio JTP, Tabungar KIB, Tengco GSC, Rio PC, Hilario AL. *Inter J Health Sci* 2020;14:52-65.	HVAC: no HVAC design features (focus on ultraviolet-C environment sterilization in hospitals)
Salonen et al [91], 2016	Salonen H, Duchaine C, Letourneau V, Mazaheri M, Laitinen S, Clifford S, Mikkola R, Lappalainen S, Reijula K, Morawska L. Endotoxin levels and contribution factors of endotoxins in resident, school, and office environments – A review. *Atmospheric Environ* 2016;42:360-69.	Study design: not a systematic review<br>Agent: not specific to virus
Saran et al [92], 2020	Saran S, Gurjar M, Baronia A, Sivapurapu V, Ghosh PS, Raju GM, Maurya I. Heating, ventilation and air conditioning (HVAC) in intensive care unit. *Crit Care* 2020;24:194.	Study design: not a systematic review<br>Agent: looks at standards to maintain good air quality, not to remove/manage virus

Scheuch [93], 2020	Scheuch G. Breathing is enough: for the spread of influenza virus and SARS-CoV-2 by breathing only. *J Aerosol Med Pulm Drug Deliv* 2020;33:230-34.	Study design: letter to the editor, not a systematic review<br>HVAC: focus on whether or not airborne transmission, not specifically about HVAC
Seto [94], 2015	Seto WH. Airborne transmission and precautions: facts and myths. *J Hosp Infect* 2015;89:225-8.	Study design: not a systematic review
Sharafi et al [95], 2020	Sharafi SM, Ebrahimpour K, Nafez A. Environmental disinfection against COVID-19 in different areas of health care facilities: a review. *Rev Environ Health* 2020;aop.	Study design: not a systematic review<br>HVAC: did not examine association of virus transmission and HVAC
Spivakovsky [96], 2020	Spivakovsky S. Which crucial measures do patients need to follow to prevent transmission of COVID-19 in the dental setting? *Evid Based Dent* 2020;21:79.	Study design: not a systematic review (commentary)
Stockwell et al [12], 2019	Stockwell RE, Ballard EL, O'Rourke P, Knibbs LD, Morawska L, Bell SC. Indoor hospital air and the impact of ventilation on bioaerosols: a systematic review. *J Hosp Infect* 2019;103:175-84.	Agent: not specific to virus
Sundell [97], 2017	Sundell J. Reflections on the history of indoor air science, focusing on the last 50 years. *Indoor Air* 2017;27:708-24.	Study design: not a systematic review<br>HVAC: did not examine association of virus transmission and HVAC
Teunis et al [98], 2010	Teunis PF, Brienen N, Kretzschmar ME. High infectivity and pathogenicity of influenza A virus via aerosol and droplet transmission. Epidemics 2010;2:215-22.	Study design: not a systematic review<br>HVAC: did not examine association of virus transmission and HVAC
Turkistani et al [99], 2020	Turkistani KA. Precautions and recommendations for orthodontic settings during the COVID-19 outbreak. *Amer J Orthod Dentofacial Orthop* 2020, In press.	Study design: not a systematic review<br>HVAC: general ventilation but not specific HVAC features
Tysiac-Mista et al [100], 2021	Tysiac-Mista M, Dubiel A, Brzoza K, Burek M, Palkiewicz K. Air disinfection procedures in the dental office during the COVID-19 pandemic. *Med Pr* 2021;72:39-48.	Study design: not a systematic review<br>HVAC: did not examine association of virus transmission and HVAC
Wang et al [101], 2020	Wang K, Wu J, Mei W, Wang X. Research status on particulate reduction technology in livestock houses. *Nongye Gongcheng Xuebao/Transactions of the Chinese Society of Agricultural Engineering* 2020;36:204-12.	Language of publication: non-English
Wu et al [102], 2018	Wu Y, Rong J, Luhung I. Influence of air conditioning and mechanical ventilation (ACMV) systems on indoor microbial aerosols. *Chin Sci Bull* 2018;63:920-30.	Language of publication: non-English
Zimmer et al [103], 2019	Zimmer C, Leuba SI, Cohen T, Yaesoubi R. Accurate quantification of uncertainty in epidemic parameter estimates and predictions using stochastic compartmental models. *Stat Methods Med Res* 2019;28:3591-608.	Study design: not a systematic review
